# Supplementary material for: Efficacy and Safety of Micropulse Transscleral Cyclophotocoagulation
Source: J Clin Med. 2022 Jun 15;11(12):3447. doi: 10.3390/jcm11123447 (PMC9224545; doi:10.3390/jcm11123447)
Supplement: Supplementary file 1 [file jcm-11-03447-s001.zip › jcm-1693233-supplementary.pdf]

Supplementary Table S1. Comparison of effect sizes and significance for intraocular pressure (mmHg) during follow-up compared to baseline using paired samples t-tests vs. a linear mixed model with repeated measurements.

**Time**	**paired t-tests**		**linear mixed model**	
	mean difference (95% CI)	p-value	mean difference (95% CI)	p-value
1 day	7.18 (5.07, 9.29)	<0.001	7.31 (4.01, 10.60)	<0.001
1 week	11.37 (8.84, 13.91)	<0.001	10.49 (7.18, 13.79)	<0.001
1 month	7.93 (6.07, 9.79)	<0.001	7.84 (4.99, 10.68)	<0.001
3 months	7.51 (5.23, 9.79)	<0.001	7.37 (3.98, 10.77)	<0.001
6 months	8.15 (6.30, 10.00)	<0.001	7.88 (5.22, 10.53)	<0.001
12 months	6.86 (4.37, 9.34)	<0.001	6.29 (3.26, 9.32)	<0.001
18 months	9.42 (6.36, 12.47)	<0.001	6.40 (2.82, 9.98)	<0.001
24 months	7.12 (3.71, 10.52)	<0.001	6.88 (3.62, 10.15)	<0.001
Last follow-up	6.20 (4.51, 7.90)	<0.001	6.23 (3.36, 9.09)	<0.001

Supplementary Table S2. Multivariate logistic regression for baseline characteristics and treatment outcome

**Trait**	OR (95% CI)	p - value
Diagnosis (primary)	1.457 (0.543 - 3.910)	0.455
Surgeon	1.402 (0.548 – 3.586)	0.480
IOP (mmHg)	0.929 (0.877 – 0.983)	0.010

Supplementary Table S3. Viability of baseline characteristics for logistic regression for treatment outcome

**Factor**	**Treatment success (n = 83)**	**Treatment failure (n = 30)**	**p-value**
Age (years)	65.7 ± 14.9	64.1 ± 13.5	0.626
Visual field mean deviation (MD; dB)	-18.5 ± 12.0	-21.3 ± 9.5	0.358
Spherical equivalent (D)**	-1.8 ± 2.9	-0.9 ± 1.3	0.115*
IOP (mmHg)	28.6 ± 10.4	21.2 ± 9.4	0.001
Diagnosis (n, %)			
- Primary glaucoma	41 (66)	21 (44)	
- Secondary glaucoma	42 (82)	9 (18)	0.052
Sex (n, %)			
- Male	45 (71)	18 (29)	
- Female	38 (76)	12 (24)	0.585
Previous surgery (n, %)			
- No surgery	27 (79)	7 (21)	
- Any surgery***	56 (71)	23 (29)	0.347
Ethnicity (n, %)			
- Caucasian	63 (71)	26 (29)	
- African	18 (90)	2 (10)	
- Asian	2 (50)	2 (50)	0.119
Surgeon (n, %)†	77	30	
- WDR	35 (65)	19 (35)	
- RCWW	42 (79)	11 (21)	0.097
Family history of glaucoma (n,%)††			
- Negative	35 (78)	10 (22)	
- Positive	20 (65)	11 (36)	0.204

* = Levene's Test significance <0.05; ** = before cataract surgery if applicable; *** = excluding cataract surgery; † = after excluding 6 patients who were treated by different physicians; †† = after excluding 37 patients for which family history was unknown.

Supplementary Table S4. Viability of baseline characteristics for logistic regression for requirement of secondary treatment

Factor	No secondary treatment required (n = 78)	Secondary treatment required (n = 36)	p-value
Age (years)	66.0 ± 14.3	63.5 ± 14.9	0.395
Visual field mean deviation (MD; dB)	-20.1 ± 11.3	-17.7 ± 11.2	0.426
Spherical equivalent (D)**	-1.6 ± 2.2	-1.4 ± 3.3	0.718
IOP (mmHg)	25.6 ± 9.6	29.1 ± 12.3	0.136*
Diagnosis (n, %)			
- Primary glaucoma	40 (65)	22 (35)	
- Secondary glaucoma	37 (71)	15 (29)	0.451
Sex (n, %)			
- Male	45 (71)	18 (29)	
- Female	32 (63)	19 (37)	0.325
Previous surgery (n, %)			
- No surgery	26 (77)	8 (23)	
- Any surgery***	51 (64)	29 (36)	0.184
Ethnicity (n, %)			
- Caucasian	59 (66)	31 (34)	
- African	15 (75)	5 (25)	
- Asian	3 (75)	1 (25)	0.680
Surgeon (n, %)†	73	35	
- RCWW	38 (69)	17 (31)	
- WDR	35 (66)	18 (34)	0.943
Family history of glaucoma (n, %)††	52	24	
- Negative	31 (69)	14 (31)	
- Positive	20 (65)	11 (35)	0.914

* = Levine's Test < 0.05; ** = before cataract surgery if applicable; *** = excluding cataract surgery; † = after excluding 6 patients who were treated by different physicians; †† = after excluding 37 patients for which family history was unknown.

Supplementary Table S5. Summary of related studies

Study	N (eyes, patients)	Total follow-up	Device settings	Baseline IOP (mmHg)*	Mean IOP reduction (mmHg, %)	Failure rate (<20% reduction)	Rate of retreatment	Rate of oral acetazolamide discontinuation	Complications
De Vries et al. (2022)	96 (84)	2 years	2000mW for 80 seconds / hemisphere if IOP <30 mmHg, 90 seconds if IOP > 30 mmHg, 31.3% duty cycle	26.6 ± 10.8	6.9 (28.1), and 7.1 after 2 years (30.2)	27%, and 32% after 2 years	38%	67.85%	None related to treatment.
De Crom et al. (2020)	141 (136)	2 years	2000mW, 2100mW for secondary treatments and 2200mW for tertiary treatments, 80 seconds / hemisphere if IOP <30 mmHg, 90 seconds if IOP > 30 mmHg, 31.3% duty cycle	23.5 ± 9.4	6.5 (27.8), and 6.7 (28.6) after 2 years	34%, and 40% after 2 years	33.30 %	70%	4.3% total: 1 (0.7%) fibrinous anterior chamber reaction, 1 (0.7%) rejection of corneal graft, 2 (1.4%) cystic macular edema, 1 (0.7%) hypotony.
Nguyen et al. (2019)	95 (95)	1 year	2000-2500mW, up to 3000mW for retreatments, 90 seconds / hemisphere, 31.3% duty cycle	25.1 ± 5.3	7.6 (30.3)	23.2%	23.20 %	Not reported	1 (1.1%) posterior synechiae, 6 (6.3%) hyphema , 3 (3.2%) persistent mydriasis, 3 (3.2%) choroidal effusion, 10 (10.5%) keratopathy, 1 (1.1%) transient hypotony.
Sarrafpour et al. (2019)	73 (62)	1 year	2500mW of only light perception or worse, 2400mW of only hand motions / counting fingers, 2250mW if VA of 20/80-20/400, 2000mW if VA of 20/20-20/70, all 50 seconds / hemisphere, 31.3% duty cycle	25.5 ± 9.4	11.7 (45.9)	24%	2.70%	73.30%	None reported.
Zaarour et al. (2019)	75 (69)	15 months	2000mW for 180 seconds total, 31.3% duty cycle	26.0 ± 7.91	8.3 (31.8)	43.3%	21%	58.13%	No serious complications were reported during follow-up.

All outcomes after 1 year of follow-up unless stated otherwise. * = mean ± standard deviation; IOP = intraocular pressure, VA = visual acuity
